# Bacteriological and physico-chemical quality of household drinking water in Kisii Town, Kisii County, Kenya

**DOI:** 10.1016/j.heliyon.2021.e06937

**Published:** 2021-05-01

**Authors:** J.K. Ondieki, D.N. Akunga, P.N. Warutere, Omanga Kenyanya

**Affiliations:** a School of Public Health, Kenyatta University, Kenya; b School of Pure and Applied Sciences, Kisii University, Kenya

**Keywords:** Safe water, Contamination, Physico-chemical, Bacteriological, Household

## Abstract

Water is a basic human need which is required in many operations especially in households. However, this essential commodity in most cases does not meet the generally accepted safety standards. The study was designed to investigate the physico-chemical and bacteriological quality of drinking water used in households in Kisii town, Kenya. Analytical cross-sectional study was conducted to obtain information concerning household drinking water quality and safety. Stratified random sampling was used to obtain 422 drinking water samples at the point of consumption from the 4 zones of Kisii town for analysis. From the study it was revealed that TDS and electrical conductivity of the analyzed water samples were within the recommended standards of less than 1000 ppm and 1500 μSCM^−1^ respectively. Further, it was found that 69.4% of the samples had pH range of between 6.5-8.5, 91.9% had turbidity of less than 5NTU, 3.8% had temperature below 15 °C and 31.2% of the chlorinated samples had chlorine residue above 0.2 ppm. In terms of bacteriological analysis, 39.3% of the samples were contaminated with total coliforms and 17.5% with *E. coli*. The main finding from the study was that the household water samples were contaminated with bacteria and unfit for human consumption because both total coliforms and *E. coli* exceeded the recommended Kenya Bureau of Standards (KEBS) and WHO standards. Therefore, public health officers should not only collect water samples from sources but also from households regularly to ascertain its quality and provide water safety promotion education to the general public. There was a strong relationship between bacterial contamination and temperature as well as chlorine residue. The study recommends Gusii Water and Sanitation Company (GWASCO) whose treatment and distribution capacity is expected to increase 4.5 times the current capacity to improve on their chlorine dosage at the treatment plant to ensure a minimum chlorine residue of 0.2 ppm at the household and community taps.

## 1 Introduction

Safe water is very important to all human beings because it is necessary for proper functioning of the body. Globally, it is approximated that 28% of the population lack access to sufficient safe water [1]. It is a scarce resource that requires utmost protection. In fact water is regarded as a basic need just like air. However, rapid population growth is a big challenge in many developing countries and this has greatly affected availability of safe water [2]. Unsafe water has been identified as a global challenge as it associated with diseases as well as chemical intoxication. Lack of safe water and poor sanitation are leading causes of preventable water borne diseases which are the world's second cause of child mortality [3]. It is estimated that globally per year, diarrhea disease causes about 502,000 deaths among young children [4].

Studies have shown that over 5 million people, majority from developing countries, die due to consumption of contaminated water and food [5]. In sub-Saharan Africa, inaccessibility of safe water is common whereby approximately 327 million people lack access to safe drinking water [3]. In the year 2012, Sub-Saharan Africa recorded about 200,000 deaths that were as a result of unsafe drinking water [4]. In Kenya alone, about 50% of diseases relate to water, Sanitation and hygiene [6].

The main sources of water in Kisii Town as per Kenya National Bureau of Statistics include spring, well, borehole and stream [7]. Most urban residents obtain their domestic water from several sources, a phenomenon commonly known as “patchwork of utilities”. In fact others obtain water from vendors without knowing the source [8]. Moreover, household water treatment (HTW) has been found to be very low especially in Kisii Town whereby more than half of the households do not practice it [9]. Treated water can also become contaminated due to improper storage and poor hygiene and sanitation practices in the households [10].

Microbial and chemical contaminants can lead to acute and chronic effects [11]. Bacterial contamination is a major health threat in relation to drinking water [12]. This contamination may occur at the source, during distribution, transportation, or due to household handling, hygiene and sanitation practices [13]. Presence of bacteria especially *E.coli* in water is an indication of pathogenic/fecal contamination [14].

Establishing drinking water quality at household is of critical importance. to ascertain its safety. Several studies have been conducted in Kisii town concerning water quality from different sources. However little has been published to show drinking water quality at the point of use in households. Given that there is limited information on physicochemical and bacteriological water quality at households in this region and many residents are unsure of safety of their drinking water at the point of use, a research of this kind is of paramount importance.

## 2 Methods

### 2.1 Study area description

**Figure 1:**
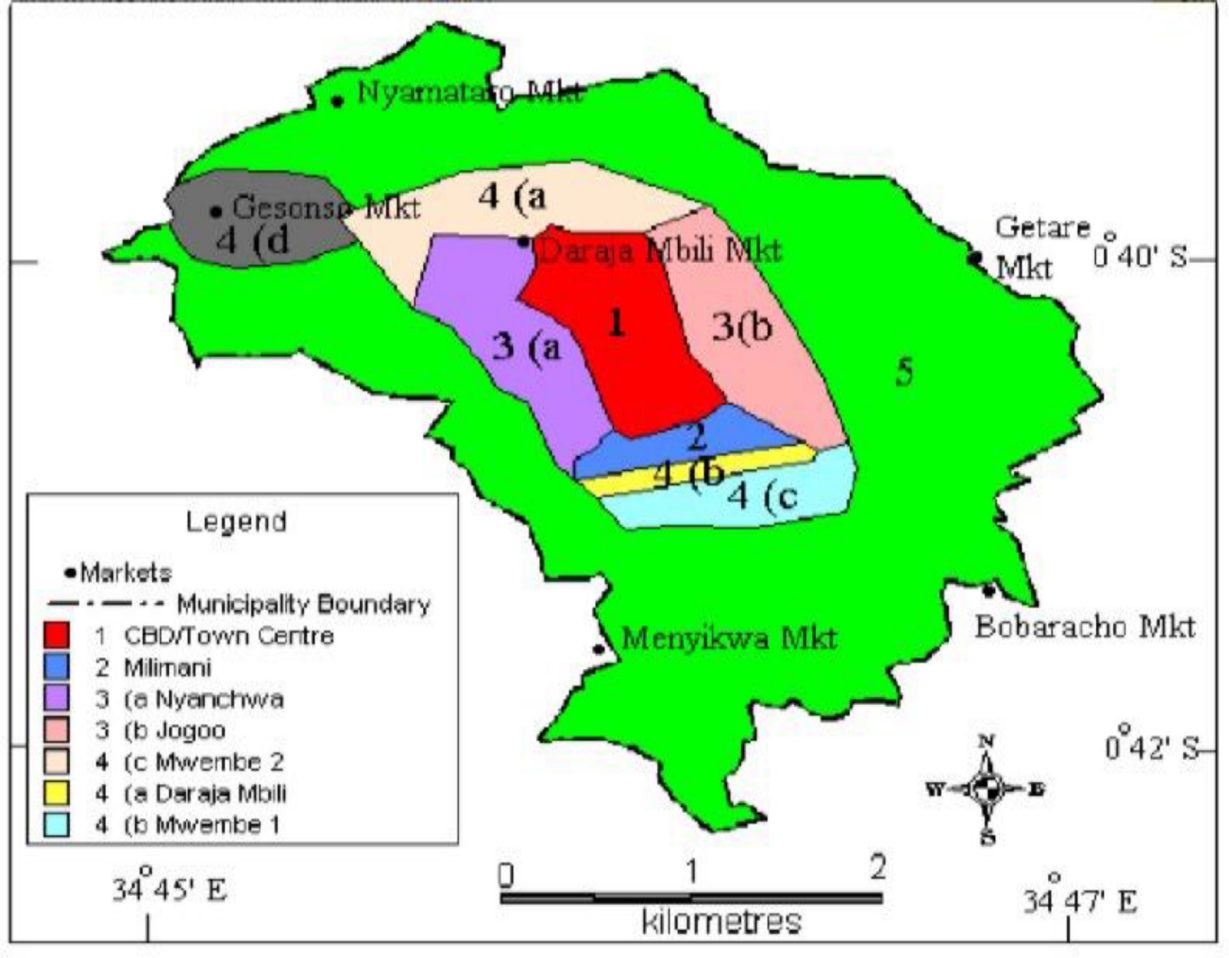
A map showing zones within Kisii Town (located within Kisii Municipality).

The study was conducted in Kisii Town located in Kisii County which is approximately about 309km North West of Nairobi which is the capital city of Kenya. Its geographical coordinates are 0° 41′ 0″ South, 34° 46′ 0″ East. The altitude is roughly 1700 m above sea level. Kisii Town is basically divided into four zones which include the CBD, Jogoo, Mwembe and Nyanchwa. Each zone has a residential estate with CBD having the least households and Jogoo having the highest. Agriculture is the major economic activity in the areas surrounding Kisii Town and also in some areas within the town. Figure 1 below is a map showing the location of the four zones of Kisii town (derived from the former municipal council).

The study employed analytical cross-sectional study. Proportionate stratified random sampling was used to obtain the 422 drinking water samples at the point of consumption for physico-chemical and bacteriological analysis. The following formula [15] was used to determine the sample size to be used as shown in the equation below. This formula is used when the population size is above 10,000.

$$\text{n}=\frac{Z^{2}PQ}{d^{2}}$$

Where,

$Z^{2}$ = Standard normal deviate. 1.96 was used in this study which corresponds to 95% level of confidence
P = Estimate proportion of an attribute that is present in the population. 0.5 was used because there was no specific proportion in this case.

Q is given by the following equation;

Q=1−P

d = Level of precision. 0.05 was used which means 95% level of confidence.

Kisii Town had a population of about 74098 by 2018 according to Kisii County Integrated Development plan 2018–2022 [16]. The following calculation was therefore used to determine the sample size:

$$n=\frac{1.96^{2}\times 0.5\times 0.5}{0.05^{2}}=384.$$

Adding 10% non-respondent rate;

384 + 38 = 422

Table 1 below illustrates how samples were selected from the four zones proportionally.

**Table 1:** Sampling frame used to select representative samples in Kisii Town.

Zone	Estimated no. of households	Estimated % of total population	Sample size
CBD	1124	6.0	25
JOGOO	11513	61.9	261
MWEMBE	4587	24.6	104
NYANCHWA	1387	7.5	32
**TOTAL**	**18611**	**100**	**422**

A pH meter with the model H1 98127 (made in Mauritius) was used to measure PH and temperature of the water samples. Turbidity was measured using a HACH 2100P portable turbidimeter (made in USA). EC and TDS were measured using EC/TDS meter model H1 99300. Free residual chlorine was determined by colorimetric method whereby DPD chlorine tablets are used [17]. Two tests were simultaneously administered to each water sample to test for total coli forms and *E. coli;* IDEXX Colilert Presence/Absence test and the 3M Petrifilm *E. coli*/ Coliform Count Plate test. The two methods were preferred because they do not depend on power supply and hence could not be affected by the rampant power shortages in the region. The petrifil plates and colilert tubes can be worn as a pouch around the waist while still providing reliable results [18]. In fact 3M petrifilm has been found to have high specificity when compared to other methods [19].

### 2.2 Study analysis and quality control

Data management and analysis was done by SPSS software version 20. Results of the studied parameters in comparison with WHO and KEBS recommendations were presented in form of tables. One-way ANOVA was carried out to establish differences in physico-chemical sample means in different strata. Chi square test was used to establish relationship between physico-chemical and bacteriological parameters.

The drinking water samples were collected at the point of use with sterilized whirl paks and were transported to the laboratory in an ice box. For piped water, the tap was opened and water was flushed for 30 s before sample collection was done. Temperature, pH and turbidity were measured in situ due to their instability. TDS, conductivity, total coli forms and *E. coli* were analyzed in the laboratory within 6 h of their collection. All the water samples were labeled precisely to avoid any form of confusion and errors [20].

## 3 Results

Various physico-chemical and bacteriological parameters were analyzed with reference to WHO and KEBS standards [21,22].

### 3.1 Physico-chemical properties of household drinking water in Kisii Town

The physico-chemical parameters that were tested include temperature, pH, turbidity, residual chlorine, TDS and electrical conductivity.

From the study, it was revealed that from the 422 of water samples analyzed, temperature range was 10.1–24.9 °C with a mean value of 22.7 °C. Sixteen (3.8%) of the samples were within WHO acceptable temperature ranges for drinking water which should not exceed 15 °C. Temperature is among the major physico-chemical parameters which are important in evaluation of household drinking water quality. It affects biological properties of water and influences performance of coagulants and disinfection products [23]. The pH of the samples had a range of 5.0–8.9 with a mean of 6.7. Majority of the samples 293 (69.4%) had WHO recommended pH of 6.5–8.5. pH is an important parameter in drinking water because it affects the effectiveness of disinfection and impacts corrosion of pipes [22]. Turbidity of the water samples ranged between 1-24 with a mean of 3.29 NTU. Most of the samples, that is, 388 (91.9%) had turbidity within the WHO recommended standards of 5NTU. Turbidity affects the aesthetic value of drinking water [24]. Chlorine residue was tested from 80 chlorinated samples obtained from households that practiced chlorination or were served by piped water. This was as per the information provided by the household head or any other adult member who gave consent. It was revealed to range from 0.04-1.6. Fifty two (65%) of these samples had residual chlorine of less than 0.2ppm, 3 (3.8%) had between 0.2-0.5ppm while 25 (31.2%) above 0.5. WHO recommends chlorine residues of between 0.2-0.5ppm especially for piped/tap water. The range for TDS was 14–71ppm with a mean value of 38.2251ppm. All the water samples were within the WHO acceptable range of less than 1000ppm. TDS affects the palatability of drinking water [25]. Electrical conductivity ranged from 27-136 μSCM^−1^ with a mean of 73.9028μSCM^−1^. All the water samples were within the acceptable range (see Table 2).

**Table 2:** Physico-chemical properties of household drinking water in Kisii Town and WHO recommended standards.

Parameters		Jogoo (N = 261)	CBD (n = 25)	Mwembe (n = 104)	Nyanchwa (N = 32)	Total (N = 422)	WHO/KEBS recommended standards
Temperature	≤15 °C (compliant)	13 (5%)	0 (%)	2 (1.9%)	1 (3.1%)	16 (3.8%)	<15 °C
	>15 °C (non-compliant)	248 (95%)	25 (100%)	102 (98.1%)	31 (96.9%)	406 (96.2%)	
	Range	10.1–24.6	21.8–24.1	12.3–24.5	22.2–24.9	10.1–24.9	
	Mean	22.	23.120	22.874	23.216	22.657	
pH	<6.5 (non- compliant)	63 (24.7%)	14 (56%)	39 (37.5%)	9 (28.1%)	125 (29.6%)	6.5–8.5
	6.5–8.5 (compliant)	195 (74.7%)	10 (40%)	65 (62.5%)	23 (71.9%)	293 (69.4%)	
	>8.5 (non-compliant)	3	1 (4%)	0 (0%)	0	4 (0.9%)	
	Range	5.8–8.9	5.0–8.6	5.7–7.9	5.8–7.8	5.0–8.9	
	Mean	6.70	6.63	6.56	6.75	6.67	
Turbidity	≤5 (compliant)	244 (93.5%)	22 (88%)	95 (91.3%)	27 (84.4%)	388 (91.9%)	<5NTU
	>5 (non-compliant)	17 (6.5%)	3 (12%)	9 (8.7%)	5 (15.6%)	34 (8.1%)	
	Range	1–11	1–16	1–24	1–8	1–24	
	Mean	3.18	3.16	3.58	3.34	3.29	
TDS	<1000 (compliant)	261 (100%)	25 (100%)	104 (100%)	32 (100%)	422 (100%)	WHO<1000ppm KBS<1500ppm
	≥1000 (non-compliant	0 (0%)	0 (0%)	0 (0%)	0 (0%)	0 (0%)	
	Range	14–64	21–54	23–61	22–71	14–71	
	Mean	36.89	37.28	40.19	43.47	38.23	
Electrical conductivity	<1000 (compliant)	261 (100%)	25 (100%)	104 (100%)	32 (100%)	422 (100%)	<1500 μSCM^−1^
	≥1000 (non-compliant)	0 (0%)	0 (0%)	0 (0%)	0 (0%)	0 (0%)	
	Range	27–132	40–103	41–118	41–136	27–136	
	Mean	71.51	72.88	77.09	83.91	73.90	
Residual chlorine		n = 43	n = 11	n = 20	n = 6	n = 80	0.2–0.5ppm
	<0.2	27 (62.8%)	9 (81.8%)	12 (60%)	4 (66.7%)	52 (65%)	
	0.2–0.5	3 (7%)	0 (0%)	0 (0%)	0 (0%)	3 (3.8%)	
	>0.5	13 (30.2%)	2 (18.2%)	8 (40%)	2 (33.3%)	25 (31.2%)	
	Range	0.05–1.42	0.05–1.1	0.04–1.6	0.09–1.1	0.04–1.6	
	Mean	0.40	0.4	0.52	0.46	0.4	

### 3.2 Comparison of physico-chemical parameters in the different zones

One way ANOVA test results indicated that there was no statistical difference between the four zones of Kisii town for temperature, pH, turbidity and chlorine residue. However, significant differences were noted for TDS and conductivity. Post Hoc test was further applied to establish the specific differences. Findings of this test revealed that TDS in Jogoo zone was 3.3ppm and 6.6ppm higher than in Mwembe and Nyanchwa respectively. The same trend was observed for electrical conductivity whereby Jogoo had 5.6 μSCM^−1^ and 12.4 μSCM^−1^ higher than Mwembe and Nyanchwa respectively. Tables 3 and 4 below shows the ANOVA and post ANOVA test results.

**Table 3:** One way ANOVA for the means of physicochemical parameters within the four zones in Kisii Town.

**Temperature**					
Source of variation	SS	Df	MS	F	p-Value
Between groups	26.53	3	8.84	1.85	0.14
Within groups	1994.67	418	4.77		
Total	2021.20	421			
**pH**					
Source of variation	SS	Df	MS	F	p-Value
Between groups	1.39	3	0.46	2.177	0.09
Within groups	88.77	418	0.21		
Total	90.16	421			
**Turbidity**					
Source of variation	SS	Df	MS	F	p-Value
Between groups	12.41	3	4.14	0.83	0.48
Within groups	2093.25	418	5.01		
Total	2105.66	421			
**TDS**					
Source of	SS	Df	MS	F	p-Value
Between groups	1769.37	3	589.79	7.05	0.001
Within groups	34994.64	418	83.72		
Total	36764.01	421			
**Conductivity**					
Source of variation	SS	Df	MS	F	p-Value
Between groups	5781.57	3	1927.19	5.96	0.001
Within groups	135109.12	418	323.23		
**Residual Chlorine**					
Source of variation	SS	Df	MS	F	p-Value
Between groups	0.27	3	0.09	0.45	0.73
Within groups	15.19	74	0.21		
Total	15.46	77

**Table 4:** Post-hoc test for means of TDS and conductivity in different zones.

	(I) zone	(J) zone	Mean difference (I-J)	significance	95% confidence interval	
					Lower bound	Upper bound
**Total dissolved Solids (TDS)**						
Turkey HSD	Jogoo	CBD	0.39	0.99	-4.55	5.33
		Mwembe	3.30	0.01	0.56	6.04
		Nyanchwa	6.58	0.001	2.16	10.99
	CBD	Mwembe	2.91	0.48	-2.35	8.17
		Nyanchwa	6.19	0.05	-0.11	12.49
	Mwembe	Nyanchwa	3.28	0.29	-1.49	8.05
**Conductivity**						
Turkey HSD	Jogoo	CBD	1.37	0.98	-8.34	11.08
		Mwembe	5.58	0.04	0.20	10.96
		Nyanchwa	12.40	0.002	3.72	21.08
	CBD	Mwembe	4.21	0.72	-6.1	14.54
		Nyanchwa	11.03	0.09	-1.34	23.40
	Mwembe	Nyanchwa	6.82	0.24	-2.55	16.19

### 3.3 Bacteriological quality of household drinking water in Kisii Town

One hundred and sixty seven (39.6%) of water samples had total coliforms and 73 (44%) of these had *E. coli.* The study findings revealed that more than half (56.3%) of the samples from Nyanchwa zone had total coliforms. Bacteriological parameters particularly total coliforms and *E coli* are commonly used in determination of general drinking water quality. Coliforms should not be present in drinking water for it to be considered safe for human consumption [26]. Table 5 below summarizes prevalence of total coliforms and *E. coli* in the four zones of Kisii town.

**Table 5:** Summary of prevalence of total coliforms and *E. coli* in different zones.

Zone	Sample Size	Total Coliforms	*E. coli*	Non *E. coli*	WHO/KEBS Standards	
					Total Coli forms	*E. coli*
Jogoo	261	101 (38.4%)	46	63	0 CFU	0 CFU
CBD	25	7 (28%)	3	4		
Nyanchwa	32	18 (56.3%)	8	10		
Mwembe	104	41 (39.4%)	16	25		
Total	422	167 (39.6%)	73	92		

### 3.4 Risk levels of drinking water with reference to bacteriological contamination

According to WHO [21], risk levels in terms of *E. coli* are categorized as shown below;

**Table:** 

Risk level	In conformity	Low risk	Intermediate risk	High risk	Very high risk
No. of colonies	0	1–10	10–100	100–1000	>1000

In the study, 348 (82.5%) of the water samples were free of *E. coli* hence in conformity with safe water requirements, 51 (12%) were in low risk category while 23 (5.5%) were in intermediate category as shown in Table 6.

**Table 6:** Risk levels of drinking water with reference to *E. coli* contamination.

*E. coli*						
Risk level (WHO, 2011)	Zone 2					
	Jogoo	CBD	Mwembe	Nyanchwa	Total	
Conformity	0CFU	214 (82 %)	22 (88 %)	88 (84.6%)	24 (75%)	348 (82.5%)
Low	1–10CFU	31 (11.9%)	1 (4%)	14 (13.5%)	5 (15.6%)	51 (12%)
Intermediate	11–100CFU	16 (6.1%)	2 (8%)	2 (1.9%)	3 (9.4%)	23 (5.5%)
High	100–1000CFU	0 (0%)	0 (0%)	0 (0%)	0 (0%)	0 (0%)
Very high	>1000CFU	0 (0%)	0 (0%)	0 (0%)	0 (0%)	0 (0%)

### 3.5 Relationship between physico-chemical properties of household drinking water and presence of total coliforms

Physico-chemical properties that had significant relationship with presence of total coliforms in household drinking water were chlorine residue (p = 0.002) and temperature (p = 0.003). Water without residual chlorine was more likely to be contaminated with total coliforms. Also, the study findings indicated that water with a temperature of more than 15 °C is more likely to be contaminated with the total coliforms. These relationships are illustrated by Table 7 below.

**Table 7:** Physico-chemical factors affecting prevalence of total coliforms in household drinking water.

Variable		Presence of total Coli forms	Absence of total Coli forms	Statistics	p-value
Chlorine residue (ppm)	Nil	150 (43.6%)	194 (56.4%)	χ^2^ = 14.79 df = 3	p-Value = 0.002
	Below 0.2	9 (17.6%)	42 (82.4%)		
	0.2–0.5	1 (33.3%)	2 (66.7%)		
	More than 0.5	6 (25%)	18 (75%)		
Temperature (^o^C)	≤15	1 (6.7%)	15 (93.3%)	χ^2^ = 7.63 df = 1 OR = 10.3	p-Value = 0.003
	>15	165 (40.6%)	241 (59.4%)		

## 4 Discussion

Temperature is among the major physico-chemical parameters important in evaluation of household drinking water quality. It affects many phenomena such as the rate of chemical reactions in water bodies such as coagulants and disinfection products, solubility of gases, amplification of tastes and colour of the water [23]. Majority (96.2%) of the temperatures recorded for the household drinking water was above WHO recommended values of less than 15 °C which makes drinking water palatable [26]. The findings are similar to the studies carried out by Yasin, *et al* [27] and Tabor, *et al* [28] in Jimma zone and Dahir Dar City in Ethiopia respectively. The high temperatures might be attributed to global warming, microbial activities and geographical location of study area (tropical temperatures) [29]. Temperature of drinking water should not exceed 15 °C as its palatability is enhanced by its coldness.

Most of the water samples (91.9%) had turbidity below 5NTU which is the maximum WHO recommended threshold for drinking water and 8.1 % had more than 5NTU. High turbidity is always associated to excess amounts of suspended organic matter and microorganisms such as bacteria. It could also be as a result of water coming into contact with surface runoffs [30]. Therefore, use of highly turbid water can be a health risk since excessive turbidity stimulates growth of pathogenic bacteria and can protect them from the effects of disinfectants [27].

The overall mean pH in the study was 6.67 with a range of 5.0–8.9. These findings are in line with those of Ogendi *et al* [31] whose range and mean pH were 5.1–9.0 and 6.76 respectively. One hundred and twenty five (29.6%) had pH below the minimum WHO accepted standards of 6.5. The low pH observed might be due to soil chemistry of the area and acid rains associated to SO_2_ and NO_X_ emissions from industries and traffic flow of vehicles which is a global problem in the recent decade [32,33]. Further, carbon IV oxide saturation can contribute to acidic nature of the water. The pH above 7 might be associated to organic materials and other chemicals [34]. Most of the water samples, 293 (69.5%) had pH within the WHO recommended values of 6.5–8.5 for potable waters. Similar findings were revealed by Yasin *et al* [27].

In the study, TDS values of the water were below the WHO and KEBS maximum allowable limits of 1000 mgL^-1^ and 1500 mgL^-1^ respectively with a mean of 38.2 mg L^−1^ thus safe for drinking. TDS include substances such as carbonates, bicarbonates, chlorides sulphates, phosphates, nitrates, calcium, magnesium, sodium and organic ions. They determine the general nature of water quality. They affect the taste of drinking water if found at high concentration above recommended values.

Electrical conductivity (EC) is the ability of any medium to conduct electrical current. Presence of dissolved substances in water such as Ca^2+^, Mg^2+^ and Cl^−^ ions carry electrical current. All EC values of the water samples were below the maximum guideline limit of 1500μSCM^−1^with a general mean of 73.9μSCM^−1^. A study by Meride and Ayenew [35] indicated similar findings with mean EC of 192.1473.9μSCM^−1^ for drinking water.

Study findings indicated that most samples obtained from piped water had residual chlorine of less than 0.2ppm which is lower than the minimum threshold recommended by WHO. On the other hand, most samples obtained from households that practiced chlorination had residual chlorine of more than 0.5ppm which is above the maximum threshold recommended by WHO. Excess chlorine in drinking water has been associated with congenital anomalies [36].

One-way ANOVA test indicated significant statistical difference in terms of TDS and conductivity in various zones of Kisii town. Jogoo zone recorded the highest readings when compared with Nyanchwa and Mwembe zones. High TDS is usually attributed to factors such as release of household water into water channels and excessive use of fertilizers from farming activities [37]. In addition to having a large residential area, Jogoo also has farms practicing modern agriculture which could have contributed to the observed results.

From the study, it was revealed that, 167 (39.3%) of the samples had total coliforms while 73 (17.3%) had *E. coli* beyond WHO acceptable levels for drinking water. In accordance to WHO guidelines, total coliforms and *E. coli* bacteria should not be detected in any water intended for drinking. Results of this study are in agreement with a recent study in Uganda by Agensi *et al* [38] where he revealed 25% of the analyzed water samples to be having total coliforms and 8.7% *E. coli*.

In the four zones studied, Nyanchwa had highest prevalence of total coliforms and *E. coli* with generally a higher risk. This could be as a result of the Nubian informal settlement found in this zone which is characterized by poor sanitation facilities with exposed excreta. Drinking water in households with exposed excreta has been found to be highly contaminated with microbial organisms [39]. The risk of acquiring a waterborne infection is positively correlated to the level of contamination by pathogenic microorganisms [26]. This study established significant relationship between bacteriological contamination of drinking water and physicochemical properties (temperature and residual chlorine). Similar findings were noted by past studies [40,41].

## 5 Conclusion

Household water in Kisii town was found to be within the WHO recommended standards with regards to TDS and EC. In terms of turbidity and pH, 91.9% and 69.4% of the drinking water samples were compliant respectively. The research revealed that 96.2% of the water samples had temperatures above maximum WHO recommended threshold of 15̊C for safe drinking water. Most water samples from households that practiced chlorination had residual chlorine exceeding the maximum WHO allowable limit of 0.5ppm. On the other hand, those from households served by piped water had less than 0.2ppm residue chlorine which is the WHO recommended minimum threshold. Bacteriological analysis indicated that 39.6% of the water samples did not meet the WHO recommended standards of 0CFU for both total coliforms and *E.coli*.

## 6 Recommendations

1. Public health officers and other relevant stakeholders should not only collect water samples from sources but also from households regularly to ascertain its bacteriological quality and provide water safety promotion education to the general public.
2. Proper water treatment should be demonstrated especially for chlorine products to ensure that people have the knowledge on correct dosage to avoid excess residual chlorine in drinking water which can be harmful to human health.
3. GWASCO that is responsible for piped water supply in Kisii Town should improve on their chlorine dosages at the treatment plant to ensure that residual chlorine at the consumer taps is always between 0.2-0.5ppm as per WHO and KEBS requirements.

## 7 Ethical approval

Ethical clearance was obtained from Kenyatta University Ethical Review Committee. A research permit was also issued by the National Commission for Science, Technology and Innovation. Permission was then granted by the Kisii County Commissioner of Education as well as Gusii Water and Sanitation Company that is responsible for the piped water serving Kisii Town. Informed consent was sought from potential participants prior to the commencement of data collection process.

## 8 Limitation of the study

Water quality is a broad subject that involves several parameters. This study focused on some selected parameters that are considered key in the determination of drinking water quality based on WHO standards. The other parameters that were not assessed in this study are as well important such as heavy metal levels.

## Declarations

### Author contribution statement

J.K. Ondieki: Conceived and designed the experiments; Performed the experiments; Analyzed and interpreted the data; Contributed reagents, materials, analysis tools or data; Wrote the paper.

D.N. Akunga and P.N. Warutere: Conceived and designed the experiments; Analyzed and interpreted the data.

O. Kenyanya: Conceived and designed the experiments; Analyzed and interpreted the data; Wrote the paper.

### Funding statement

This research did not receive any specific grant from funding agencies in the public, commercial, or not-for-profit sectors.

### Data availability statement

The data that has been used is confidential.

### Declaration of interests statement

The authors declare no conflict of interest.

### Additional information

No additional information is available for this paper.
